# Increased Trimethylation of histone H3K36 associates with biliary differentiation and predicts poor prognosis in resectable hepatocellular carcinoma

**DOI:** 10.1371/journal.pone.0206261

**Published:** 2018-10-24

**Authors:** Huang-Chun Lien, Yung-Ming Jeng, Yu-Ling Jhuang, Ray-Hwang Yuan

**Affiliations:** 1 Department of Pathology, National Taiwan University Hospital, Taipei, Taiwan; 2 Department of Pathology, College of Medicine, National Taiwan University, Taipei, Taiwan; 3 Graduate Institute of Pathology, College of Medicine, National Taiwan University, Taipei, Taiwan; 4 Department of Integrated Diagnostics & Therapeutics, National Taiwan University Hospital, Taipei, Taiwan; 5 Department of Surgery, National Taiwan University Hospital, Taipei, Taiwan; 6 Department of Surgery, College of Medicine, National Taiwan University, Taipei, Taiwan; University of Navarra School of Medicine and Center for Applied Medical Research (CIMA), SPAIN

## Abstract

**Introduction:**

Trimethylation of histone H3K36 (H3K36me3), an epigenetic marker of transcription-associated histone modification and stem cell regulation, is expressed in a variety of human cancers. This study elucidated the prognostic significance of H3K36me3 in patients with resectable hepatocellular carcinoma (HCC).

**Methods:**

Expression of H3K36me3 was retrospectively evaluated through immunohistochemistry in 152 surgically resected primary HCCs.

**Results:**

In nontumorous liver parenchyma, H3K36Me3 was detected in bile ducts but not in hepatocytes. H3K36me3 was positive in 104 (68.4%) of the HCCs. Positivity for H3K36me3 was associated with high level of serum α-fetoprotein (>200 ng/mL, *P* = 0.0148), high tumor grade (*P* = 0.0017), and high tumor stage (*P* = 0.0008). Patients with H3K36me3-positive tumors were more likely to have lower 5-year disease-free survival and 5-year overall survival than those with H3K36me3-negative tumors (*P* = 0.0484 and *P* = 0.0213, respectively). Multivariate analysis showed that H3K36me3 positivity was an independent predictor of high tumor grade (*P* = 0.0475) and high tumor stage (*P* = 0.0114) and thus contributed to poor prognosis. Furthermore, H3K36me3 positivity was significantly correlated with the expression of biliary markers cytokeratin 19 (CK19) and hepatocyte nuclear factor 1β (*HNF1β*) (*P* < 0.0001 and *P* = 0.0005, respectively). Combinatorial analysis revealed that CK19 and *HNF1β* expression individually exerted additive prognostic adverse effects on HCCs with H3K36me3 positivity.

**Conclusions:**

Our study indicates that H3K36me3 positivity is associated with the expression of biliary markers and is a crucial predictor of poor prognosis in resectable HCC.

## Introduction

Hepatocellular carcinoma (HCC) is one of the leading malignancies worldwide. The incidence of HCC is higher in some parts of the world, particularly in sub-Saharan Africa, southern China, Southeast Asia, and Taiwan. Although HCC is currently less common in developed Western countries, the incidence of HCC is increasing there as well [1]. The main risk factors for HCC are viral hepatitis B infection, viral hepatitis C infection, aflatoxin exposure, and liver cirrhosis of various etiologies [2]. Although surgical resection plays a crucial role for cancer treatment and other tumor ablation methods can be introduced for local tumor treatment, the survival of HCC patients remains poor because of high rates of intrahepatic tumor recurrence and extrahepatic metastasis [3]. Molecular studies have indicated frequent mutations in *p53* and *β-catenin* genes [4–6]. Somatic mutations of other genes are infrequent. Therefore, studies about epigenetic and gene expression changes are mandatory for the understanding of molecular factors contributing to progression and poor prognosis of HCC; such understanding is required to establish effective therapeutic targets and treatment strategies.

Trimethylation of histone H3K36 (H3K36me3), an epigenetic marker associated with actively transcribed genes [7], is proposed to be involved in numerous biological processes, such as DNA mismatch repair [8, 9], chromatin structure modulation during elongation [10], and stem cell regulation [11]. Loss of function mutations of the tumor suppressor SET domain containing 2 (*SETD2*) [12], overexpression of oncogene lysine demethylase 4A (*KDM4A*) [13], or mutation of histone H3.3 can decrease H3K36me3 in cancer [14]. Although the alternation of H3K36me3 has been discovered in various malignant tumors [15, 16] and the role of H3K36me3 in hepatitis B virus X protein-mediated gene regulation has been noted [17], the clinicopathological significance of H3K36me3 status in human HCC remains unclear.

In this study, we used immunostaining to analyze H3K36me3 expression in a large cohort of patients with HCC in Taiwan and analyzed the correlation of H3K36me3 expression with clinicopathological and molecular factors.

## Materials and methods

### Tissue samples

For this retrospective study, 152 patients with surgical resectable, primary unifocal HCC diagnosed within the period from April 1993 to October 2004 at National Taiwan University Hospital (NTUH) were selected. The patients comprised 122 men and 30 women, and all of them had received comprehensive pathological assessment and regular follow-up examinations, as described elsewhere [18–21]. The mean age of the patients was 54.9 years (range, 14–88 years). The study procedures were compliant with ethical guidelines, and all participants provided written consent under the regulation of the National Taiwan University Hospital Research Ethics Committee (approval no. 201602017RINC). One 14-year-old boy was enrolled in our study, whose parents provided written informed consent. The anonymity of all patients was protected; all specimens were evaluated blindly. Both serum hepatitis B surface antigen (HBsAg) and hepatitis C antibodies (anti-HCVs) were positive in 14 patients; 111 were positive for only HBsAg, and 39 were positive for only anti-HCVs. Each patient had satisfactory liver function reserve at the time that the patient’s operation was performed. None of these patients had distant metastasis before surgery. No patients were receiving anticancer therapies such as percutaneous ethanol injection, radiofrequency ablation, transhepatic arterial chemoembolization, or chemotherapy before surgery.

### Histology and tumor staging

Surgically resected, formalin-fixed, paraffin-embedded HCC specimens were cut into 5-μm thick sections and then stained with hematoxylin and eosin. Tumor grade was divided into three groups based on the criteria proposed by Edmondson and Steiner [22]. Tumor staging was identified as stages I (56 cases), II (34 cases), and III (62 cases) on the basis of the criteria of the 8th version of American Joint Committee on Cancer system [23]. Patients with HCC categorized as stage IVA and IVB were excluded because we emphasized the prognostic significance of surgically resectable tumors in this study. In addition, tumor margins were checked microscopically, and patients with incomplete tumor resection were excluded.

### Immunohistochemical detection of H3K36me3, cytokeratin 19 (CK19), and hepatocyte nuclear factor 1β (*HNF1β*)

Archival formalin-fixed, paraffin-embedded sections of nontumorous liver tissue and HCC specimens were used to analyze the expression of H3K36me3, CK19, and *HNF1β* using the streptavidin-biotin immunoperoxidase technique, as described previously [19, 21]. The primary antibodies used were a rabbit polyclonal antibody against human H3K36me3 (1:100 dilution, Abcam, Cambridge, MA, USA), a mouse monoclonal antibody against human CK19 (1:200 dilution, Leica Biosystems, Newcastle, UK), and a rabbit polyclonal antibody against human *HNF1β* (1:100 dilution; Sigma-Aldrich, St. Louis, MO, USA). For negative controls, the primary antibodies were replaced with 5% fetal bovine serum. Additionally, the hepatocytes and bile ducts from patients with liver hemangioma and uninfected livers were used as negative and positive controls, respectively. For representativeness, homogeneity, and fairness, the percentages of immunostaining-positive cells were calculated on five independent microscopic fields of each slide under 400× magnification by one pathologist who was unaware of the outcome of any patients. For data presentation, the percentage of cancer cells that was positive for H3K36me3 immunostaining was categorized using 4 degrees of positivity. Diffuse expression was defined as expression of H3K36me3 in more than 50% of tumor cells. Heterogeneous or focal expression was defined as expression of H3K36me3 in 11%–50% of tumor cells, and expression of H3K36me3 in 1%–10% tumor cells was defined as small-proportion expression; these were regarded as the H3K36me3-positive group. In the nontumorous liver, H3K36me3 was detected in the bile duct and only in a few isolated liver cells. Therefore, HCCs with immunopositivity for H3K36me3 in less than 1% of tumor cells were regarded as the negative group. The expressions of CK19 protein and *HNF1β* protein were considered positive if staining was seen in ≥5% of the tumor cells, as described previously [18, 19].

### Detection of *p53* and *β-Catenin* mutations

Mutation of the *p53* gene was detected in 119 cases by direct DNA sequencing on the region spanning exon 2 to exon 11, as described previously [4]. Mutation of *β-catenin* was detected in 127 cases through direct sequencing of DNA on the exon 3 region [24].

### Follow-up examination and management

All of the 152 patients received follow-up for over 5 years or until death, whichever happened earlier. Among all patients, 45 (29.6%) survived for more than 5 years, and 132 (86.8%) were suitable for tumor recurrence evaluation. During follow-up in outpatient clinics, patients received assessment of serum α-fetoprotein (AFP) at 1- to 2-month intervals and abdominal ultrasonography surveillance of liver at 3-month intervals. In patients with clinical suspicions of tumor recurrence, magnetic resonance imaging or computed tomography was requested to prove intrahepatic recurrence or distal metastasis. All patients with tumor recurrence were evaluated for the possibility of operative reresection. However, those with extrahepatic metastasis, portal vein, inferior vena cava tumor invasion, poor liver function, or other comorbidity that complicated the surgery were excluded from operative reresection. Patients with small recurrent lesions (≤5 cm in diameter) that could be accessed through percutaneous radiofrequency ablation (RFA) or would have been dangerous if treated by reresection were treated by RFA. Patients with recurrent lesions that could not be removed completely or patients who refused to receive the aforementioned therapeutics received transhepatic arterial chemoembolization if serum total bilirubin <2.0 mg/dL. Patients who were unable or unwilling to receive any of the aforementioned management treatments were treated through support treatment.

### Statistical analysis

StatCalc for Windows (Epi Info Version 7.2, Centers for Disease Control and Prevention, Atlanta, GA, USA) was used for data analysis. The χ^2^ test and Fisher’s exact tests were used for univariate analysis. The Kaplan–Meier estimate of survival was used for the cumulative survival rates after tumor resection, and differences between survival curves were evaluated using the log-rank test. Multivariate analyses of factors correlated with tumor grade and tumor stage were conducted with logistic regression models, and factors correlated with 5-year survival were analyzed by Cox’s proportional-hazard regression models. All the parameters used in univariate analyses were used in multivariate analyses. A two-tailed *P* < 0.05 was considered statistically significant.

## Results

### H3K36me3 in liver and HCC

**Fig 1:**
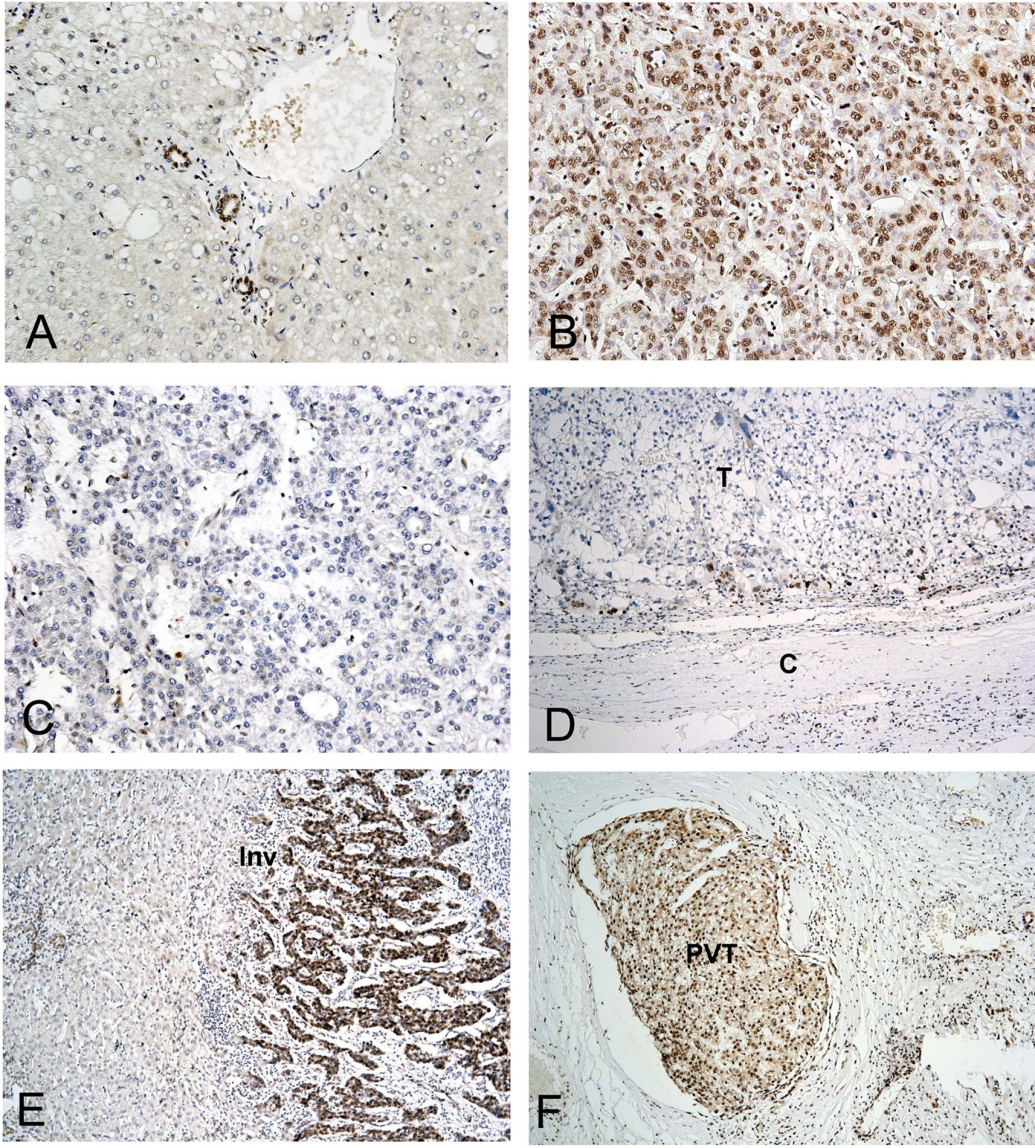
Immunostaining of H3K36me3 in normal liver parenchyma and HCC. (A) H3K36me3 detected in bile duct epithelial cells in the portal area but not in the hepatocytes or mesenchymal cells of normal liver tissue. (B) Diffuse nuclear staining of H3K36me3 in HCC. (C) Absence of immunostaining of H3K36me3 in HCC. (D) H3K36me3 immunostaining was more prominent at the periphery of the tumor mass near the tumor capsule. (E) Strong H3K36me3 immunostaining along the invasion front of noncapsulated HCC. (F) Portal vein tumor embolus exhibiting strong H3K36me3 immunostaining. T: tumor, C: capsule, Inv: invasion front, PVT: portal vein tumor embolus. A–C 200×, D–F 100× (original magnification).

In the nontumorous liver parenchyma, H3K36me3 was detected in the nuclei of bile duct epithelial cells in the portal areas but not in the liver mesenchymal cells or hepatocytes (Fig 1A). H3K36me3 was detected in the nuclei of tumor cells in 104 of 152 HCCs (68.4%), with diffuse expression in 29 cases (19.1%; Fig 1B), nodular or heterogeneous expression in 44 (28.9%), and focal expression in 31 (20.4%); these were grouped together as the H3K36me3-positive group. The other 48 tumors with negative staining or positive staining in fewer than 1% of tumor cells were regarded as the H3K36me3-negative group (31.6%; Fig 1C). Using this categorization, we found that the proportion of H3K36me3-positive patients with HCC gradually increased with the tumor stage (51.8% in stage I, 76.5% in stage II, 75.0% in stage IIIA, and 81.0% in stage IIIB; *P* = 0.0064). In tumors with heterogeneous staining, the staining was predominantly located at the peripheries of the tumors (Fig 1D) and along the invasion fronts (Fig 1E). It is noteworthy that the intravascular tumor thrombi in portal vein branches often exhibited intense and diffuse immunoreactivity (Fig 1F).

### Clinicopathological significance of H3K36me positivity in HCC

To clarify the effect of H3K36me3 positivity in HCC, we analyzed relationships of positive H3K36me3 immunostaining with major clinical and pathological features. As shown in Table 1, H3K36me3 positivity in HCCs was significantly associated with high serum α-fetoprotein level (AFP >200 ng/mL, *P* = 0.0148). However, it was uncorrelated with age, gender, status of HBsAg, and anti-HCV status. Histologically, H3K36me3 positivity exhibited a borderline association with large tumor size (>5 cm, *P* = 0.0542) and a significant association with high tumor grade (grade III–IV, *P* = 0.0017) but not with liver cirrhosis, *p53* mutation, or *β-catenin* mutation. Notably, patients with high-stage (stage II–III) HCCs had significantly higher H3K36me3 levels compared with those with low-stage HCCs (stage I, *P* = 0.0008).

**Table 1:** Analysis of H3K36Me3 expression with various clinicopathological features in 152pateints with surgically resectable primary hepatocellular carcinoma.

		H3K36Me3 expression				
Variables		Total	No n(%)	Yes n (%)	O.R. (95% C.I.)	*P* value
**Age**	**≤ 56**	**77**	**21**	**56 (72.7)**	**1.0**	
	**> 56**	**75**	**27**	**48 (64.0)**	**1.32 (0.65–2.67)**	**0.4039**
**Gender**	**Male**	**122**	**41**	**81 (66.4)**	**1.0**	
	**Female**	**30**	**7**	**23 (76.7)**	**1.66 (0.61–4.67)**	**0.2781**
**HBsAg**	**(—)**	**41**	**15**	**26 (63.4)**	**1.0**	
	**(+)**	**111**	**33**	**78 (70.3)**	**1.36 (0.60–3.099)**	**0.4197**
**Anti-HCV**	**(—)**	**100**	**30**	**70 (70.0)**	**1.0**	
	**(+)**	**39**	**15**	**24 (61.5)**	**0.697 (0.30–1.60)**	**0.3381**
**AFP (ng/ml)**	**≤ 200**	**74**	**30**	**42 (56.8)**	**1.0**	
	**> 200**	**78**	**18**	**60 (76.9)**	**2.38 (1.11–5.13)**	**0.0148**
**Cirrhosis**	**(—)**	**105**	**32**	**73 (69.5)**	**1.0**	
	**(+)**	**47**	**16**	**31 (66.0)**	**0.85 (0.38–1.89)**	**0.6620**
**Tumor size (cm)**	**≤ 5**	**62**	**25**	**37 (60.0)**	**1.0**	
	**> 5**	**90**	**23**	**67 (74.4)**	**1.97 (0.93–4.18)**	**0.0542**
**Tumor grade**	**I-II**	**96**	**39**	**57 (59.4)**	**1.0**	
	**III-IV**	**56**	**9**	**47 (83.9)**	**3.57 (1.48–8.86)**	**0.0017**
**Tumor stage**	**I**	**56**	**27**	**29 (51.8)**	**1.0**	
	**II-III**	**96**	**21**	**75 (78.1)**	**3.08 (1.54–7.24)**	**0.0008**
***p53* mutation**	**(—)**	**64**	**22**	**42 (65.6)**	**1.0**	
	**(+)**	**55**	**17**	**38 (69.1)**	**1.17 (0.51–2.72)**	**0.6880**
***β-catenin* mutation**	**(-)**	**113**	**37**	**76 (67.3)**	**1.0**	
	**(+)**	**14**	**5**	**9 (64.3)**	**0.88 (0.24–3.27)**	**0.9376**
**CK19**	**(-)**	**87**	**40**	**47 (54.0)**	**1.0**	
	**(+)**	**56**	**6**	**50 (89.3)**	**7.09 (2.57–20.59)**	**< 0.0001**
***HNF1β***	**(-)**	**45**	**23**	**22 (48.9)**	**1.0**	
	**(+)**	**70**	**14**	**56 (80.0)**	**4.18 (1.48–4.42)**	**0.0005**

Abbreviations: O.R., Odds ratio; C.I., Confidence interval; AFP, α-fetoprotein; CK19, cytokeratin 19

HNF1β, hepatocyte nuclear factor 1β.

(-) designates absence; (+) designates presence.

### H3K36me3 positivity predicts tumor recurrence and poor prognosis in HCC

**Fig 2:**
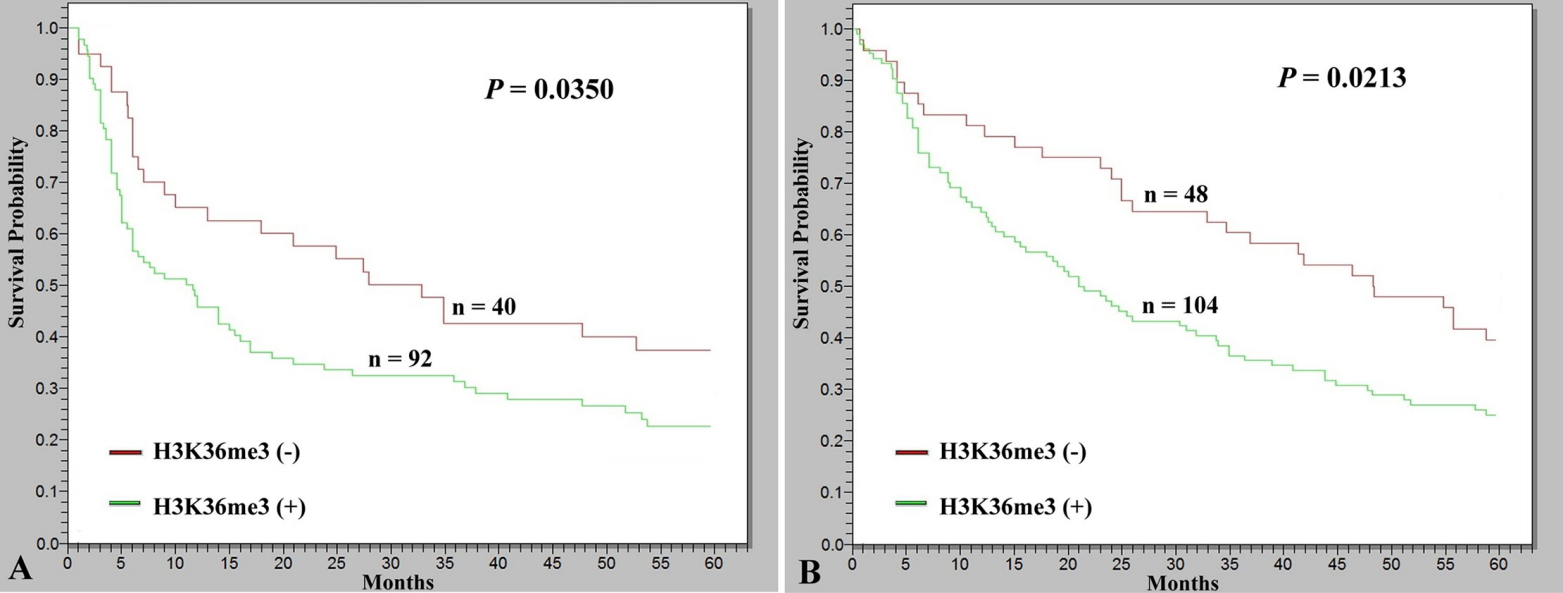
Kaplan–Meier analysis of 5-year disease-free survival and 5-year overall survival in HCC patients. Patients with H3K36me3-positive HCC had significantly lower disease-free survival than those with H3K36me3-negative HCC (*P* = 0.0350). (B) Patients with H3K36me3-positive HCC had significantly lower overall survival than those with H3K36me3-negative HCC (*P* = 0.0213).

In this study’s cohort, tumors recurred within 5 years in 104 of 132 patients (78.8%). Patients with H3K36Me3-positive HCCs had significantly higher incidence of tumor recurrence than those with H3K36me3-negative tumors (77/92 versus 27/40, *P* = 0.0365). Furthermore, a Kaplan-Meier survival curve estimated that patients with HCC who were H3K36me3-positive had a lower 5-year disease-free survival rate than did those who were H3K36me3-negative (Fig 2A; *P* = 0.0350).

To identify independent factors predicting patient survival, we employed all the clinicopathological parameters analyzed in univariate analysis and H3K36me3 status in multivariate analyses. As Table 2 shows, presence of serum HBsAg was an independent predictor of high tumor grade (*P* = 0.0056). High serum AFP level and absence of β*-catenin* mutation were independent predictors of high tumor stage (*P* = 0.0030 and *P* = 0.0283, respectively). Notably, H3K36me3 positivity was identified as an independent predictor of high tumor grade and high tumor stage (*P* = 0.0475 and *P* = 0.0114, respectively), and therefore contributed to poor prognosis with lower 5-year overall survival than for those without H3K36me3 positivity (Fig 2B; *P* = 0.0213).

**Table 2:** Multivariate analyses of the risk factors associated with tumor grade, tumor stage, and survival of the patients with surgically resectable primary hepatocellular carcinoma.

Covariate	Coefficient	S.E.	Z-Statistic	O.R./H.R. (95% C.I.)	*P* value
Grade*					
**Age (L/H)**	**-0.8925**	**0.5072**	**-1.7598**	**0.0496 (0.1516–1.1069)**	**0.0784**
**Gender (M/F)**	**-0.9709**	**0.6295**	**-1.5423**	**0.3787 (0.1103–1.3008)**	**0.1230**
**Serum HBsAg (P/N)**	**2.0003**	**0.7222**	**2.7697**	**7.3914 (1.7947–30.4417)**	**0.0056**
**Anti-HCV (P/N)**	**0.7637**	**0.6219**	**1.2280**	**2.1462 (0.6343–7.2619)**	**0.2195**
**AFP (L/H)**	**-0.3774**	**0.5289**	**-0.7134**	**0.6857 (0.2432–1.9336)**	**0.4756**
**Cirrhosis (N/Y)**	**-0.4044**	**0.6150**	**-0.6576**	**0.6674 (0.2000–2.2276)**	**0.5108**
**Tumor Size (S/L)**	**-1.0687**	**0.5610**	**-1.9050**	**0.3434 (0.1144–1.0313)**	**0.0568**
***p53* mutation (P/N)**	**0.7167**	**0.4992**	**1.4356**	**2.0476 (0.7697–5.4473)**	**0.1511**
***β-catenin* mutation (P/N)**	**0.2930**	**0.8103**	**0.3616**	**1.3404 (0.2739–6.5604)**	**0.7177**
**H3K36me3 expression (P/N)**	**1.0678**	**0.5388**	**1.9819**	**2.9091 (1.0119–8.3633)**	**0.0475**
Stage*					
**Age (L/H)**	**1.0659**	**0.6097**	**1.7483**	**2.9034 (0.8790–9.5904)**	**0.0804**
**Gender (M/F)**	**1.0687**	**0.7811**	**1.3682**	**2.9116 (0.6299–13.4586)**	**0.1712**
**Serum HBsAg (P/N)**	**-0.4054**	**0.7423**	**-0.5461**	**0.6667 (0.1556–2.8563)**	**0.5850**
**Anti-HCV (P/N)**	**-0.7226**	**0.7369**	**-0.9806**	**0.4855 (0.1145–2.0579)**	**0.3268**
**AFP (L/H)**	**-1.8610**	**0.6279**	**-2.9636**	**0.1555 (0.0454–0.5325)**	**0.0030**
**Cirrhosis (N/Y)**	**-0.7414**	**0.7624**	**-0.9724**	**0.4764 (0.1069–2.1232)**	**0.3308**
**Tumor Size (S/L)**	**-1.1781**	**0.6218**	**-1.8948**	**0.3079 (0.0910–1.0414)**	**0.0581**
**Grade (L/H)**	**-0.9166**	**0.6440**	**-1.4233**	**0.3999 (0.1132–1.4129)**	**0.1547**
***p53* mutation (P/N)**	**1.1467**	**0.6311**	**1.8170**	**3.1479 (0.9137–10.8444)**	**0.0692**
***β-catenin* mutation (P/N)**	**-2.0414**	**0.9311**	**-2.1926**	**0.1298 (0.0209–0.8053)**	**0.0283**
**H3K36me3 expression (P/N)**	**1.5274**	**0.6034**	**205312**	**4.6064 (1.4116–15.0317)**	**0.0114**
**Survival time**^**‡**^					
**Age (L/H)**	**0.0674**	**0.2709**	**0.2489**	**1.0698 (0.6290–1.8193)**	**0.8034**
**Gender (M/F)**	**-0.3836**	**0.3105**	**-1.2357**	**0.6814 (0.3708–1.2521)**	**0.2166**
**Serum HBsAg (P/N)**	**-0.2593**	**0.3686**	**-0.7036**	**0.7716 (0.3747–1.5889)**	**0.4817**
**Anti-HCV (P/N)**	**0.4520**	**0.3362**	**1.3445**	**1.5714 (0.8131–3.0368)**	**0.1788**
**AFP (L/H)**	**0.1640**	**0.2927**	**0.5602**	**1.1782 (0.6638–2.0911)**	**0.5753**
**Cirrhosis (N/Y)**	**-1.6301**	**0.3714**	**-4.3888**	**0.1959 (0.0946–0.4057)**	**<0.0001**
**Tumor Size (S/L)**	**-1.3648**	**0.3704**	**-3.6846**	**0.2554 (0.1236–0.5279)**	**0.0002**
**Grade (L/H)**	**-0.1916**	**0.2958**	**-0.6478**	**0.8256 (0.4624–1.4742)**	**0.5171**
**Stage (L/H)**	**1.8254**	**0.3787**	**4.8205**	**6.2056 (2.9542–13.0353)**	**<0.0001**
***p53* mutation (P/N)**	**-0.1376**	**0.2648**	**-0.5195**	**0.8715 (0.5186–1.4644)**	**0.6034**
***β-catenin* mutation (P/N)**	**-0.2002**	**0.4831**	**-0.4145**	**0.8185 (0.3176–2.1097)**	**0.6785**
**H3K36me3 expression (P/N)**	**-0.3380**	**0.2889**	**-0.1170**	**0.7132 (0.4048–1.2564)**	**0.2420**

Abbreviations: S.E., Standard error; O.R., Odds ratio; H.R., Hazard ratio; C.I., Confidence interval; M, male; F, female; AFP, α-fetoprotein; H3K36me3,trimethylation of histone H3K36; L, low or large; H, high; P, presence; N, absences or no; Y, yes; S, small.

*Logistic regression model

^‡^Cox’s proportional hazards model

### Combinatorial analyses of H3K36me3 positivity with CK19 expression in HCC tumor progression

CK19 is reported to be a cell marker of biliary [25] and progenitor cells [26], and is expressed throughout the biliary system in the adult liver [25]. In this study, H3K36me3 immunopositivity was not only detected in HCC tissues but also in the bile ducts of noncancerous liver parenchyma. As shown in Table 1, a strong correlation of H3K36me3 positivity with CK19 expression was demonstrated (*P* < 0.0001). Therefore, we further analyzed the role of H3K36me3 positivity in CK19 expression individually.

**Fig 3:**
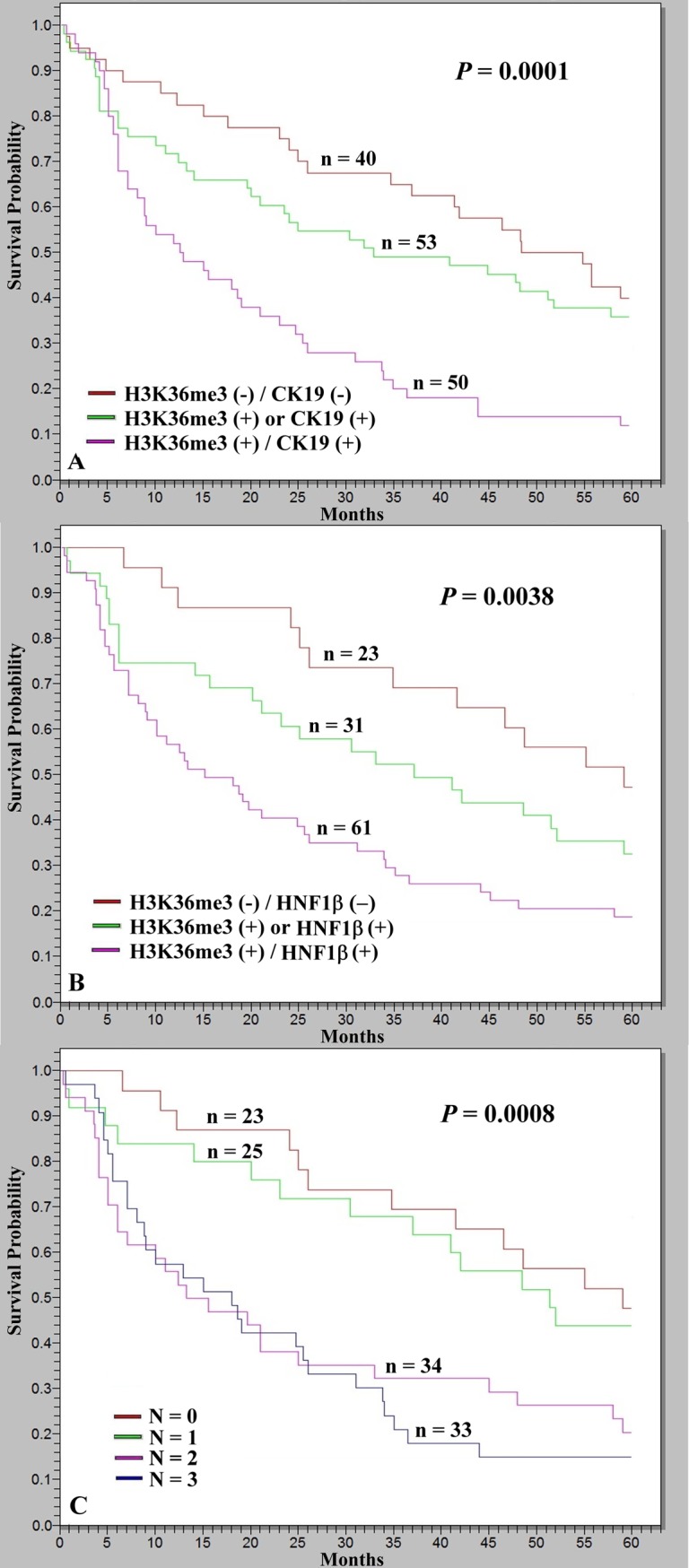
Kaplan–Meier analysis of cumulative 5-year overall survival (OS) in relation to the number of presented markers in HCC patients. (A) Patients with HCCs who were positive for both H3K36me3 and CK19 expression exhibited the lowest 5-year OS, significantly lower than that of patients with HCCs with either H3K36me3 positivity or CK19 expression (*P* = 0.0027), whereas patients with HCCs without H3K36me3 positivity or CK19 expression had the highest 5-year OS (*P* = 0.0001). (B) Patients with HCCs who were positive for both H3K36me3 and *HNF1*β expression had the lowest 5-year OS, marginally lower than those with HCCs with either H3K36me3 positivity or *HNF1*β expression (*P* = 0.0530), whereas patients with HCCs without H3K36me3 positivity or *HNF1*β expression had the highest 5-year OS (*P* = 0.0038). (C) Cumulative survival in relation to the number of presented markers (H3K36me3, CK19, and *HNF1*β). Patients presenting more markers were more likely to exhibit lower 5-year OS (*P* = 0.0008); however, the differences in survival of patients with HCC positive for two or three markers were nonsignificant. N, number of markers present.

As listed in Table 3, combinatorial analysis revealed that 103 of 143 tumors (72.0%) expressed at least one of the two markers, H3K36me3 or CK19. HCCs with simultaneous positivity for H3K36me3 and CK19 exhibited the highest incidences of high serum AFP level, high tumor grade, and high tumor stage (*P* = 0.0002, *P* = 0.0001, and *P* < 0.0001, respectively). HCCs positive for either H3K36me3 or CK19 alone had lower incidences, and the incidences were lowest in HCCs negative for both markers. A Kaplan–Meier survival curve estimated that the patients with HCC who were positive for both H3K36me3 and CK19 had the lowest 5-year survival rate, which was significantly lower than that of patients with HCC who were positive for either CK19 or H3K36me3 alone (*P* = 0.0027). The patients with HCC who were negative for CK19 and H3K36me3 had the highest 5-year survival rate (Fig 3A; *P* = 0.0001).

**Table 3:** Combinatorial analyses of H3K36me3 expression with CK19 expression or *HNF1*β expression in the tumor progression of hepatocellular carcinoma.

Feature	N = 0 n (%)	N = 1 n (%)	N = 2 n (%)	*P* value
**H3K36me3/CK19**				
**AFP (ng/ml)**				
**≤ 200**	**27 (67.5%)**^**a**^	**25 (47.2%)**	**17 (34.0%)**^**a**^	**0.0002**
**>200**	**13 (32.5%)**	**28 (52.8%)**	**33 (66.0%)**	
**Grade**				
**I-II**	**35 (87.5%)**^**b**^	**35 (66.0%)**^**c**^	**22 (44.0%)**^**b**^^,^^**c**^	**0.0001**
**III-IV**	**5 (12.5%)**	**18 (34.0%)**	**28 (56.0%)**	
**Stage**				
**I**	**25 (62.5%)**^**d**^^,^^**e**^	**21 (39.6%)**^**e**^^,^^**f**^	**7 (14.0%)**^**d**^^,^^**f**^	**<0.0001**
**II-III**	**15 (37.5%)**	**32 (60.4%)**	**43 (86.0%)**	
**H3K36me3/*HNF1β***				
**AFP (ng/ml)**				
**≤ 200**	**16 (69.6%)**^**g**^	**14 (45.2%)**	**19 (31.1%)**^**g**^^,^	**0.0061**
**>200**	**7 (30.4%)**	**17 (54.8%)**	**42 (68.9%)**	
**Grade**				
**I-II**	**21 (91.3%)**^**h**^^,^^**i**^	**20 (64.5%)**^**i**^	**36 (59.0%)**^**h**^	**0.0384**
**III-IV**	**2 (8.7%)**	**11 (35.5%)**	**25 (41.0%)**	
**Stage**				
**I**	**13 (56.5%)**^**j**^	**11 (35.5%)**	**18 (29.5%)**^**j**^	**0.0715**
**II-III**	**10 (43.5%)**	**20 (64.5%)**	**43 (70.5%)**	

Abbreviations: H3K36me3, Trimethylation of histone H3K36; CK19, cytokeratin 19; HNF1β, Hepatocyte nuclear factor 1β; AFP, α-fetoprotein.

a: 0.0016

b: <0.0001

c: 0.0245

d: <0.0001

e: 0.0289

f: 0.0035

g: 0.0014

h: 0.0047

i: 0.0228

j: 0.0221.

N = 0, designates none of the two genes expressed; N = 1, designates either of the two genes expressed; N = 2, designates both of the genes expressed.

### Combinatorial analyses of H3K36me3 positivity with HNF1β expression in HCC tumor progression

Hepatocyte nuclear factor 1β (*HNF1*β) is a regulator of biliary differentiation and strongly expressed in the biliary system of the adult liver [27]. As shown in Table 1, H3K36me3 positivity demonstrated a strong association with *HNF1*β expression (*P* = 0.0005). Therefore, it is valuable to elucidate the relationship of H3K36me3 positivity with *HNF1*β expression. As listed in Table 3, combinatorial analysis revealed that 92 of 115 tumors (80.0%) expressed at least one of the two markers, H3K36me3 or *HNF1*β. Patients with HCCs who were positive for both H3K36me3 and *HNF1*β had the highest incidence of high serum AFP level and high-grade tumor (*P* = 0.0061 and *P* = 0.0384, respectively) as well as borderline association with high-stage tumor (*P* = 0.0715). Patients with HCCs who were positive for either H3K36me3 or *HNF1*β alone had lower incidences, and the incidences were lowest in patients with HCCs who were negative for H3K36me3 and *HNF1*β. A Kaplan–Meier survival curve estimated that the patients with HCC who were positive for both H3K36me3 and *HNF1*β had the lowest 5-year survival rate and a marginally lower rate than patients with HCC who were positive for either H3K36me3 or *HNF1*β (*P* = 0.0530). Patients with HCC who were negative for H3K36me3 and *HNF1*β had the highest 5-year survival rate (Fig 3B; *P* = 0.0038).

### Combinatorial analysis of H3K36me3, CK19, and *HNF1*β expression in the progression of HCC

As aforementioned, CK19 and *HNF1*β expression individually exerted additive prognostic adverse effects on HCCs with H3K36me3 positivity. Further combinatorial analysis was conducted to elucidate the roles of these three markers in progression of HCC. In this study, 115 patients had all of the three examined markers, and 92 cases (80.0%) had at least one marker. Therefore, we categorized the HCC patients into four groups based on the number of markers expressed: *N* = 0 (no marker expressed), *N* = 1 (any one of the three markers expressed), *N* = 2 (any two of the three markers expressed), and *N* = 3 (all three markers expressed). Patients with HCCs expressing more than one marker were more likely to exhibit high serum AFP levels, high tumor grades, and high tumor stages (Table 4; *P* = 0.0014, *P* = 0.0208, and *P* = 0.0007, respectively). Importantly, a Kaplan–Meier survival curve estimated that HCC patients with positivity for more markers had a lower 5-year survival rate (Fig 3C; *P* = 0.0008).

**Table 4:** Combinatorial analysis of H3K36me3 signature enrichment, CK19 expression and HNF1β expression in the tumor progression of hepatocellular carcinoma.

Feature	Number of events H3K36me3/CK19/HNF1β				
	N = 0 n (%)	N = 1 n (%)	N = 2 n (%)	N = 3 n (%)	*P* value
**AFP (ng/ml)**					
**≤ 200**	**16 (69.6)**^**a**^^,^^**b**^	**14 (56.0)**	**10 (29.4)**^**a**^	**9 (26.5)**^**b**^	**0.0014**
**>200**	**7 (30.4)**	**11 (44.0)**^**c**^^,^^**d**^	**24 (70.6)**^**c**^	**24 (72.7)**^**d**^	
**Grade**					
**I-II**	**21 (91.3)**^**e**^^,^^**f**^	**19 (76.0)**	**19 (55.9)**^**e**^	**18 (54.5)**^**f**^	**0.0208**
**III-IV**	**2 (9.5)**	**6 (24.0)**	**15 (44.1)**	**15 (45.4)**	
**Stage**					
**I**	**13 (56.5)**^**g**^^,^^**h**^	**15 (60.0)**^**i**^	**8 (23.5)**^**g**^	**6 (18.2)**^**h**^^,^^**i**^	**0.0007**
**II-III**	**10 (43.5)**	**10 (40.0)**	**26 (76.5)**	**27 (81.8)**	

Abbreviations: H3K36me3, Trimethylation of histone H3K36; CK19, cytokeratin 19; HNF1β, Hepatocyte nuclear factor 1β; AFP, α-fetoprotein.

a: 0.0013

b: 0.0017

c: 0.0216

d: 0.0268

e: 0.0101

f: 0.0081

g: 0.0113

h: 0.0029

i: 0.0010.

N = 0, designated none of the three genes expressed; N = 1, designated any one of the three genes expressed; N = 2, designated any two of the three genes expressed; N = 3, designated all of the three genes expressed.

## Discussion

Epigenetic alterations are influential in cancer initiation and progression [28]. DNA methylation, histone modification, and long noncoding RNA are major forms of epigenetic regulations, affect chromatin structure and gene transcription, and result in the activation of oncogenes or inactivation of tumor suppressor genes [29, 30]. Histone modification through methylation or acetylation causes changes in the structure of chromatin and can be used as a marker of such changes [29]. Trimethylation of H3 lysine 36 (H3K36me3) and H3 lysine 4 (H3K4me3) is tightly related to active gene transcription, whereas trimethylation of H3 lysine 9 (H3K9me3) and H3 lysine 27 (H3K27me3) is related to repressed gene transcription [31]. Therefore, we were interested in elucidating the role of H3K36me3 in HCC.

In this study, H3K36me3 was detected through immunohistochemistry in 68.4% of the 152 HCCs; furthermore, H3K36me3 positivity in patients with HCC was significantly correlated with high serum AFP level. In addition, multivariate analysis confirmed the independent predictive role of H3K36me3 positivity in HCC tumor grade. Interestingly, H3K36me3 expression was more obvious in the peripheral regions of tumor borders, invasive fronts, and tumor thrombi in the portal branches, suggesting the possible role of H3K36me3 in tumor invasion and metastasis. This finding is consistent with the report by Dominguez et al. that H3K36me3 was highly expressed on a set of genes that promote cell proliferation [32].

One mechanism of regulation of gene expression by H3K36me3 is directing the usage of splicing donor sites. For example, higher H3K36me3 levels induces increased skipping of the final 83 base pairs of *CDH1* exon 8, resulting in cell discohesion, which may be an underlying mechanism of a relatively high tumor stage in H3K36me3-positive HCC [33]. Increased H3K36me3 level has also been reported in other types of cancer; H3K36me3 was increased in 58 of the 67 core set genes with robust periodic expression in multiple cell types, which associated with shortened survival when upregulated in breast tumors [32]. Yang et al. reported that overexpression of *NSD2*-mediated NF-κB-activation-associated elevation of histone H3K36me3 marks in prostate cancer [34]. Increased H3K36me3 level was also reported in breast cancer [35]. Taken together, these findings suggest that increased H3K36me3 level may induce active genetic transcription in cancer-promoting genes to facilitate tumor progression; however, the molecular targets of H3K36me3 in HCC require further research.

The overexpression of H3K36me3 in cancer seems to be contradictory to the loss of function mutations of *SETD2* and loss of expression of H3K36me3 in renal cell carcinoma and high-grade glioma [15, 16]. According to the COSMIC database, the incidences of both truncating and missense mutations of SETD2 in HCC were very low (truncating mutation 0.78%, missense mutation 1.12%) [36]. Besides, the same epigenetic markers may have different targets and play different roles in different cell types. A more well-known example is about trimethylated histone H3 on lysine 27 (H3K27me3) in cancer. Overexpression of H3K27me3 caused by activating mutation or overactivity of Enhancer of zeste homolog 2 (EZH2), a histone methyltransferase and a catalytic component of polycomb repressive complex 2, is a frequent event in many types of cancer [37]. In contrast, complete loss of H3K27me3, caused by either inactivation of EED or SUV12, was detected in vast majority of malignant peripheral nerve sheath tumors [38].

Although surgical resection is a potentially curative treatment, the survival of patients with surgically resected HCCs remains low mainly because of high tumor recurrence rates [3, 4]. Therefore, appropriate markers must be identified for prediction of tumor recurrence and development of improved patient management strategies. In this study, we demonstrated for the first time that patients with HCC who have positive H3K36me3 expression are at significantly higher risk of tumor recurrence and, thus, have lower 5-year disease-free survival than those with negative H3K36me3 expression. HCCs with positive H3K36me3 expression exhibit aggressive behaviors, such as occult tumor metastasis or vascular invasion, leading to a higher incidence of tumor recurrence and, consequently, lower disease-free survival and poor prognosis in patients after tumor resection. Therefore, immunostaining for H3K36me3 can be used to identify a crucial molecular predictor for patients with HCC who are at elevated risk of recurrence after tumor resection.

Because H3K36me3 was detected in the biliary epithelium but hepatocytes were not detected in nontumorous liver parenchyma, and because our published studies indicated that HCC with biliary differentiation is associated with poor prognosis [18, 19], we were interested in the association of H3K36me3 positivity with biliary markers. We found a strong association between H3K36me3 positivity and biliary marker expression. In cases with heterogeneous or focal staining, H3K36me3 positivity was predominantly identified in the tumor periphery, invasion front, and tumor emboli, where biliary markers commonly were expressed. This observation suggests possible roles of H3K36me3 in the regulation of biliary differentiation. Combinatorial analyses revealed that expression of these markers contributed to poor prognosis. H3K36me3 is probably enriched in the loci of genes regulating biliary differentiation, both in nontumorous liver tissue and liver cancers, inducing a biliary differentiation program.

The major limitations of this study are that we did not provide any evidence for the mechanism of H3K36me3 positivity in hepatocarcinogenesis. Although H3K36me3 might indicate the active transcription of the biliary markers, CK19 and *HNF1*β, we also did not elucidate the mechanism of H3K36me3 involvement in biliary marker expression. In addition, the superior prediction of H3K36me3 positivity over other immunohistochemical markers remains to be clarified.

In conclusion, our study showed that H3K36me3 positivity is a critical independent predictor of high tumor grade and high tumor stage and contributes to tumor recurrence and poor prognosis in HCC. Combinatorial analysis revealed that CK19 and *HNF1*β expression individually exerted additive prognostic adverse effects on HCCs with H3K36me3 positivity. The findings also emphasized the importance of biliary differentiation in HCC progression. Thus, H3K36me3 may serve as a new target of gene therapy for HCC.
